# Liraglutide Inhibits Endothelial-to-Mesenchymal Transition and Attenuates Neointima Formation after Endovascular Injury in Streptozotocin-Induced Diabetic Mice

**DOI:** 10.3390/cells8060589

**Published:** 2019-06-14

**Authors:** Tzu-Hsien Tsai, Chien-Ho Lee, Cheng-I Cheng, Yen-Nan Fang, Sheng-Ying Chung, Shyh-Ming Chen, Cheng-Jei Lin, Chiung-Jen Wu, Chi-Ling Hang, Wei-Yu Chen

**Affiliations:** 1 Division of Cardiology, Department of Internal Medicine, Kaohsiung Chang Gung Memorial Hospital and Chang Gung University College of Medicine, Kaohsiung 83301, Taiwan; garytsai@adm.cgmh.org.tw (T.-H.T.); gentolata@cgmh.org.tw (C.-H.L.); chris.chengi.cheng@gmail.com (C.-I.C.); wideopen@cgmh.org.tw (Y.-N.F.); miosheny@cgmh.org.tw (S.-Y.C.); Syming99@gmail.com (S.-M.C.); cjlin4@gmail.com (C.-J.L.); cvcjwu@cloud.cgmh.org.tw (C.-J.W.); samuelhang@hotmail.com (C.-L.H.); 2 Institute for Translational Research in Biomedicine, Kaohsiung Chang Gung Memorial Hospital, Kaohsiung 83301, Taiwan

**Keywords:** endothelial-mesenchymal transition, hyperglycaemia, liraglutide, neointima formation

## Abstract

Hyperglycaemia causes endothelial dysfunction, which is the initial process in the development of diabetic vascular complications. Upon injury, endothelial cells undergo an endothelial-to-mesenchymal transition (EndMT), lose their specific marker, and gain mesenchymal phenotypes. This study investigated the effect of liraglutide, a glucagon-like peptide 1 (GLP-1) receptor agonist, on EndMT inhibition and neointima formation in diabetic mice induced by streptozotocin. The diabetic mice with a wire-induced vascular injury in the right carotid artery were treated with or without liraglutide for four weeks. The degree of neointima formation and re-endothelialisation was evaluated by histological assessments. Endothelial fate tracing revealed that endothelium-derived cells contribute to neointima formation through EndMT in vivo. In the diabetic mouse model, liraglutide attenuated wire injury-induced neointima formation and accelerated re-endothelialisation. In vitro, a high glucose condition (30 mmol/L) triggered morphological changes and mesenchymal marker expression in human umbilical vein endothelial cells (HUVECs), which were attenuated by liraglutide or Activin receptor-like 5 (ALK5) inhibitor SB431542. The inhibition of AMP-activated protein kinase (AMPK) signaling by Compound C diminished the liraglutide-mediated inhibitory effect on EndMT. Collectively, liraglutide was found to attenuate neointima formation in diabetic mice partially through EndMT inhibition, extending the potential therapeutic role of liraglutide.

## 1. Introduction

Diabetes mellitus (DM) causes hyperglycaemia and systemic inflammation, leading to endothelial dysfunction [1,2,3]. Endothelial damage is the initial process in the progression of diabetic vascular complications [4,5]. Vascular complications are the major causes of mortality and morbidity in patients with DM [4,5]. Endothelial-to-mesenchymal transition (EndMT) is the process by which endothelial cells lose their specific markers and acquire mesenchymal or myofibroblastic phenotypes [6,7]. The endothelial origin of mesenchymal or fibroblast-like cells can be characterized by the loss of cell-cell junctions, acquisition of invasive and migratory capacities, loss of endothelial markers (e.g., CD31, Tie2, Tie1, Vascular endothelial (VE)-Cadherin, and von Willebrand factor (vWF)), and gain of mesenchymal markers (e.g., fibroblast-specific protein 1(FSP-1), alpha-smooth muscle actin (a-SMA), smooth muscle-22alpha actin (SM22α), N-cadherin, and vimentin) [7,8,9,10].

EndMT plays a vital role in cardiovascular diseases, such as cardiomyopathy, pulmonary hypertension, atherosclerosis, and neointimal hyperplasia [6,9,10,11,12]. Interestingly, recent studies have also revealed that inflammation and diabetic conditions, such as high glucose, trigger the trans-differentiation of endothelial cells into mesenchymal-like cells [13,14,15,16]. Therefore, the EndMT process may be the initial stage of neointima hyperplasia under diabetic conditions. 

Liraglutide is a long-acting analogue of a glucagon-like peptide 1 (GLP-1) receptor agonist and is used for lowering blood glucose in patients with diabetes [17]. Liraglutide is also believed to possess cardiovascular protective effects in patients with diabetes [17]. Recently, a GLP-1 agonist has been found to attenuate atherosclerotic lesions and endothelial function by inhibiting the inflammation process [18,19]. Liraglutide-mediated AMP-activated protein kinase (AMPK) activation has been linked to amelioration of palmitate-induced endothelial dysfunction [20]. In addition, the GLP-1 analogue was shown to protect against hyperglycaemia-induced EndMT [21,22] and myocardial dysfunction by suppressing poly (Adenosin diphosphoribose) polymerase 1 activity [21]. However, it is unclear whether the liraglutide-mediated inhibition of EndMT is involved in the neointima hyperplasia, occurring after a vascular injury in diabetic conditions. In this study, we examined the impact of liraglutide on high glucose-induced EndMT both in vitro and in vivo in the context of neointima formation in a diabetic condition.

## 2. Materials and Methods

### 2.1. Animals

All animal experiments were conducted in accordance with the Guide for the Use and Care of Laboratory Animals and were approved by the local Institutional Animal Care and Use Committee (IACUC) committee of Kaohsiung Chang Gung Memorial Hospital, Kaohsiung, Taiwan. (IACUC number: 2015092201). Wild type C57BL/6 mice were purchased from the National Laboratory Animal Center (NLAC), Taipei, Taiwan. C57BL/6-Tg (Tek-RFP, cre/ERT2) 27Narl (RMRC13162, GEMMS NLAC, Taiwan) mice were crossed with C57BL/6J-Tg (UBC-DsRed-emGFP) 22Narl (RMRC13119, GEMMS NLAC, Taipei, Taiwan) mice to obtain double transgenic mice (TekCreERT2/DRG). The genotypes of these mice were confirmed using PCR with specific primers: GFP-forward: TGAACCGCATCGAGCTGAAGGG; GFP-reverse: TCCAGCAGGACCATGTGATCGC; Cre-forward: CTAAACATGCTT CATCGTCGGTC; Cre-reverse: TCTGACCAGAGTCATCCTTAGCG. The 5-week-old double transgenic mice (TekCreERT2/DRG) were treated with intraperitoneal (i.p.) tamoxifen (Sigma–Aldrich, St. Louis, MO, USA) (1 mg/20 g mouse/day; dissolved in corn oil) for 5 consecutive days to induce the Cre-mediated deletion of the floxed DsRed allele. The recombined allele expresses green fluorescence protein (GFP) in Tek-expressing cells. After a recovery period of at least 2 weeks after the first tamoxifen injection, the double transgenic mice (TekCreERT2/DRG) were subjected to streptozotocin (STZ)-induced diabetes.

### 2.2. Streptozotocin (STZ)-Induced Diabetic Mouse Model

Daily i.p. STZ injections (50 mg/kg) were used to induce diabetic conditions in 9-week-old male C57BL/6 mice and/or double transgenic (TekCreERT2/DRG) mice weighing 18–20 g for 5 consecutive days. The fasting serum glucose level was checked every week until all mice were considered diabetic (blood glucose exceeded 250 mg/dL for 3 consecutive readings). Wire injury-induced endothelial denudation surgery was performed in these diabetic mice when they reached 12 weeks of age (1 week after the diabetic condition was confirmed). Mouse tail vein blood was used to test for the serum glucose level using One Touch Ultra (LifeScan; Johnson & Johnson, New Brunswick, NJ, USA) on day 0, 14, and 28.

### 2.3. Endothelial Denudation and Measurement of Neointima Formation

To investigate the impact of liraglutide on neointima formation in vivo, twenty 12-week-old male diabetic C57BL/6 mice were anaesthetized with xylazine hydrochloride (16 mg/kg body weight, Rompun^®^ 2%, Bayer, Leverkusen, Germany) and i.p. Zoletil (0.01 mL/g). Endothelial denudation of the right common carotid artery was performed as previously described with modification [23]. Briefly, the right internal common carotid artery was isolated near the carotid bifurcation. The right common carotid artery was denuded and dilated using a straight guide wire (0.16 mm in diameter) that was inserted approximately 15 mm from the internal carotid artery near the bifurcation site with three rotational passes through a transverse arteriotomy of the internal carotid artery. All mice recovered, and no symptoms of stroke were observed after this procedure. Animals received a daily i.p. injection of liraglutide (1000 μg/kg, once daily) [24] or normal saline (same volume) in the control group for 28 days. In addition, mice in the control group would receive 0.1 U insulin per day if their serum glucose levels exceeded 500 mg/dL. After 4 weeks, the animals were euthanized, and the isolated arteries were fixed with 4% paraformaldehyde in phosphate-buffered saline (PBS) via left ventricle injection. The areas of the right common carotid arteries, 20 mm around the bifurcation site, were paraffin-embedded and sectioned for ten continuous serial slides (5 μm in thickness) for haematoxylin and eosin (H and E) staining. Images were captured using a bright-light microscope (Olympus BX51, Tokyo, Japan). A morphometric analysis was performed using Image J software. Measurements were performed at a magnification of 200× with the image analysis software (Image J). The intimal area was calculated as the area inside the internal elastic lamina-luminal area, while the medial area was calculated as the area encircled by the external elastic lamina area inside the internal elastic lamina; these calculations were done separately for each artery. The percentage of intimal expansion of the arterial segment in each section was calculated with the following formula: Intimal expansion % = (I/M) × 100%; where the intima area I corresponds to the area within the internal elastic lamina, and M was the medial area.

Age-matched mice in the control group were sacrificed at the same time. To investigate the EndMT process in neointima formation, 12-week-old diabetic double transgenic mice (TekCreERT2/DRG) underwent the same procedure and were sacrificed 4 weeks after the right common carotid artery endothelial denudation surgery. The tissues of the right common carotid arteries were also collected using the methods mentioned above.

### 2.4. Re-Endothelialisation Assay 

The area of neo-endothelisation was evaluated by denuded areas stained with 100 μL of 5% Evans blue dye via penis vein injection, as modified from a previously published study [25]. The extravasations of the Evans blue dye from the compromised endothelial gap junctions into the surrounding tissue represented the loss of endothelial integrity and poor re-endothelisation following vascular damage. The re-endothelialised area was calculated by the planimetric quantification of the ratio of the unstained area (re-endothelialised area) and the Evan blue-stained area (injured surface area), using ImageJ analysis software (image processing and analysis in JAVA; http://rsb.info.nih.gov/ij/) version 1.43.

### 2.5. Cell Culture

Primary human umbilical vein endothelial cells (HUVECs) were purchased from the Bioresource Collection and Research Center (BCRC), Hsinchu, Taiwan. Briefly, cells were cultured in a M199 medium containing 5% fetal bovine serum (FBS), 1% endothelial cell growth factors (02-102, Millipore, Burlington, MA, USA), and 1% penicillin/streptomycin at 37 °C with 5% CO_2_. HUVECs between 3 to 6 passages were cultured in monolayer dishes for the experiment. At approximately 80% confluence, the culture medium was changed to a serum-free medium for 24 h before the cells were used for further experiments. 

### 2.6. Induction of EndMT in HUVECs In Vitro

To examine the effect of high glucose on the phenotypic transition in HUVECs, cells were treated with normal glucose (NG: 5.5 mM), high glucose (HG: 30 mM d-glucose), and an iso-osmotic medium containing mannitol (5.5 mM NG + 24.5 mM mannitol) for 96 h. Cells were harvested for further analyses of EndMT markers using qRT-PCR, western blotting, and immunofluorescence staining. To examine the effect of liraglutide or Activin receptor-like 5 (ALK5) inhibition on HG- and IL-1β-induced EndMT, the HUVECs were treated with PBS, HG (30 mM), IL-1β (10 ng/mL), HG (30 mM) + IL-1β (10 ng/mL), HG (30 mM) + IL-1β (10 ng/mL) + liraglutide (1 μg/mL), HG (30 mM) + IL-1β (10 ng/mL) + liraglutide (1 μg/mL) + SB431542 (1 μM), or HG (30 mM) + IL-1β (10 ng/mL) + SB431542 (1 μM) for 24 h. Cells were harvested for the western blot analysis of TGF-β, p-Smad2, and p-AMPK.

### 2.7. Histology and Immunofluorescence Staining

For cell immunofluorescence staining, HUVECs were fixed in 4% paraformaldehyde for 20 min and then permeabilized in 0.03% Triton X-100 for 15 min. After blocking in 5% bovine serum albumin for 30 min at room temperature, cells were incubated overnight at 4 °C with primary antibodies against SM22α (1:1000; Ab14106, Abcam, Cambridge, UK) and VE-cadherin (1:500; sc-6458, Santa Cruz Biotechnology, Santa Cruz, CA, USA) and then with fluorescent dye-conjugated secondary antibodies (Thermo Fisher Scientific, Waltham, MA, USA). Nuclei were visualized with 4′,6-diamidino-2-phenylindole·2HCl (DAPI) (Santa Cruz Biotechnology).

For tissue immunofluorescence staining, mouse carotid arteries were fixed with 4% paraformaldehyde, paraffin embedded, sectioned, and stained by standard immunohistochemistry and fluorescent microscopy methods, as previously described [26]. An additional antigen retrieval step was applied by heating samples in a Tris-based buffer (pH 9.0) to 95 °C for 20 min. After blocking in 5% bovine serum albumin for 30 min at room temperature, tissues were incubated overnight at 4 °C with primary antibodies against SM22α (1:1000; ab14106, Abcam), α-SMA (1:1000; ab5694, Abcam), VE-cadherin (1:500; sc-6458, Santa Cruz Biotechnology), or GFP (1:200; SC-9996, Santa Cruz Biotechnology) and then with fluorescent dye-conjugated secondary antibodies (Thermo Fisher Scientific). The endothelium was labelled with biotin-conjugated isolectin B4 (IB4) (1:100; I21414, Invitrogen, Carlsbad, CA, USA) and a streptavidin-Alexa Fluor™ 647 conjugate (1:500; S21374, Thermo Fisher Scientific). Nuclei were visualized with DAPI. Images were captured using an immunofluorescence microscope (Olympus BX51, Tokyo, Japan) or an automated confocal laser-scanning microscope (Olympus FV10i).

### 2.8. Quantitative Real-Time qPCR

Total RNA was extracted using a GENEzolTM TriRNA pure kit (Geneaid, Taipei City, Taiwan) according to the user manual. Complementary DNA (cDNA) was synthesized from 1 μg of total RNA and random hexamers using the Transcriptor First Strand cDNA Synthesis kit (Roche, Basel, Switzerland). Forward and reverse primers were designed from sequences of different exons of the target gene to avoid the amplification of genomic DNA. One microliter of diluted cDNA was used per 20 μL reaction using the QuantiNova TM SYBR Green PCR kit (QIAGEN, Hilden, Germany). Real-time PCR was performed on a StepOnePlus real-time PCR system (Applied Biosystems, Foster City, CA, USA), with 40 cycles of 95 °C for 5 s and 60 °C for 1 min. All reactions were performed in triplicate. Details of the primer sequences used are presented in Table S1.

### 2.9. Western Blotting

Cell lysates from cultured HUVECs were extracted using a radioimmunoprecipitation assay buffer (RIPA) buffer (150 mM NaCl, 0.1% SDS, 0.5% sodium deoxycholate, 1× protease inhibitor cocktail (Roche), and 50 mM Tris; pH 8.0), and the total protein concentration of cell extracts was determined using a bicinchoninic acid assay (BCA) protein assay kit (Pierce, Thermo Fisher Scientific). After resolution using sodium dodecyl sulfate (SDS) polyacrylamide gels, poly(vinylidene fluoride) (PVDF) membrane filter papers were used for immunoblotting. The washing buffer comprised 0.1% Tween 20 in Tris-buffered saline (20 mM Tris and 150 mM NaCl; pH 7.4), and the blocking buffer was 5% skim milk (Bio-Rad, Hercules, CA, USA) in a washing buffer. EndMT-related proteins including TGF-β (1:1000; GTX110630, GeneTex, Irvine, CA, USA), SM22α (1:10,000; ab14106, Abcam), Snail (1:500; C15D3, Cell Signaling Technology, Danvers, MA, USA), CD31 (1:1000; 89C2, Cell Signaling Technology), total Smad2/3 (1:1000; D7G7, Cell Signaling Technology), phospho-Smad2 (1:500; Ser465/467; Cell Signaling Technology), vimentin (1:10,000; ab92547, Abcam), total AMPK (1:5000; #2532, Cell Signaling Technology), and phospho-AMPK (1:5000; 40H9, Cell Signaling Technology) were examined.

### 2.10. Statistical Analysis

All data are presented as mean ± standard deviation (SD). Data were analyzed using Student’s t-tests or Kruskal–Wallis non parametric test. *P* < 0.05 was considered statistically significant. Statistical analyses were performed using Statistical Product and Service Solutions (SPSS) 18.0 (SPSS Inc., Chicago, IL, USA).

## 3. Results

### 3.1. The GLP-1 Agonist Liraglutide Improves Re-Endothelialisation and Reduces Neointima Lesions after Arterial Injury in Diabetic Mice

**Figure 1:**
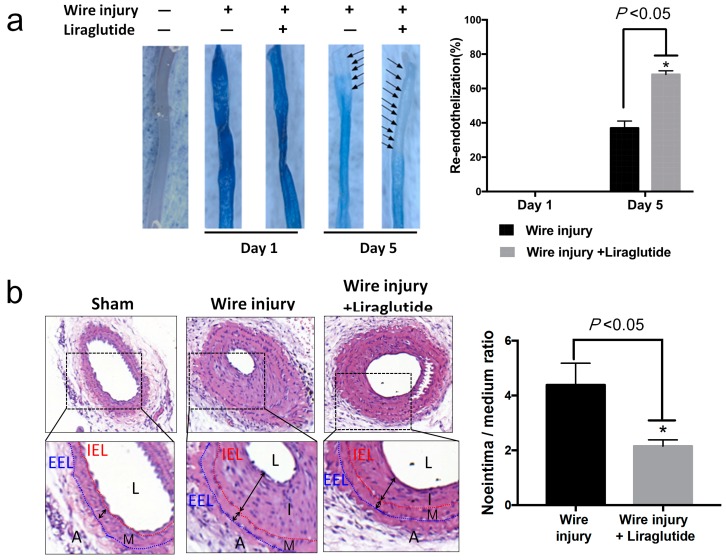
Liraglutide promotes endothelium recovery and reduces neointima formation in wire-injured carotid arteries of diabetic mice. Streptozotocin (STZ)-induced diabetic C57BL/6 mice were subjected to a wire-induced carotid artery injury with or without liraglutide treatment. (**a**) The percentage of the area of re-endothelisation was determined after the systemic injection of Evans blue at day one and day five (*n* = 3 per group). The stained blue area corresponds to the area with poor re-endothelialisation. The black arrowheads point at the area without blue staining area, which indicated re-endothelialised cells. Data are shown as mean ± SEM. * *P* < 0.05 analyzed by Student’s *t*-test. (**b**) The right common carotid arteries were collected on day 28 post-injury for haematoxylin and eosin (H and E) staining to evaluate the severity of neointima formation between groups. Data are shown as the mean ± SD (all groups, *n* = 10 per group), * *P* < 0.05 analyzed by Student’s *t*-test. IEL, internal elastic lamina; EEL, external elastic lamina; L, lumen; I, intima; M, media; A, adventitia.

Liraglutide, a GLP-1 agonist, has been used for treating diabetes and is proven to have a cardiovascular protective effect [17,27]. To evaluate whether liraglutide improves endothelial function and post-injury endothelium recovery, we performed a wire injury in the carotid artery of streptozotocin (STZ)-induced diabetic C57BL/6 mice with or without liraglutide treatment. Vascular re-endothelisation was quantified by Evans blue staining of the denuded area at one and five days after injury (Figure 1a). Endothelium was severely damaged after injury and recovered by 37% on day five in the wire-injury group without liraglutide treatment. On day one, there was no difference between the two groups with and without liraglutide treatment. However, diabetic mice with liraglutide treatment displayed a higher percentage of re-endothelisation when compared to mice without liraglutide treatment (day five: Liraglutide 68% versus Control 37%, *P* < 0.05) (Figure 1a). 

A glycaemic analysis showed higher serum glucose levels in mice following STZ-treatment and confirmed the induction of a diabetic condition, whereas no significant difference was observed between the wire-injured groups with and without liraglutide treatment (Table S2). Wire injury-induced neointima hyperplasia was observed in STZ-induced diabetic mice at 28 days post-injury (Figure 1b). A cross-sectional analysis showed a decreased neointima-to-media ratio in the liraglutide-treated group compared with the control group (Figure 1b). These results indicate liraglutide improves endothelium recovery and reduces neointima formation in diabetic mice. 

### 3.2. Endothelial Fate-Mapping Model

**Figure 2:**
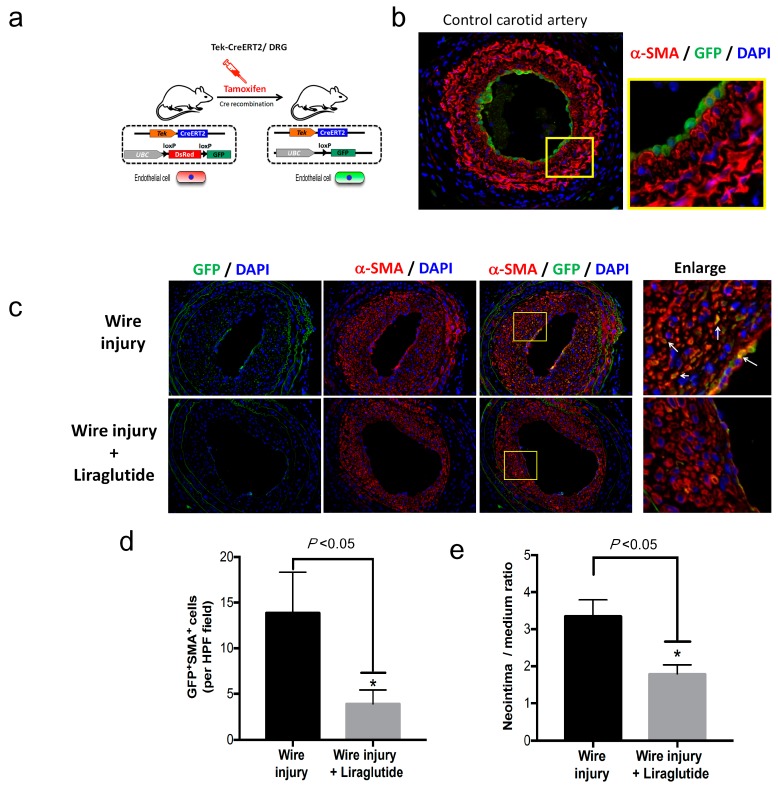
Endothelia-derived cells contribute to neointima formation through EndMT. (**a**) Schematic representation of the induction of endothelial-specific GFP labelling after tamoxifen injection in TekCreERT2/DRG mice. (**b**) Representative images of immunofluorescence staining for the protein expression of GFP (green) and α-SMA (red) in the common carotid artery. Nuclei were counterstained with 4′,6-diamidino-2-phenylindole·2HCl (DAPI) (blue). The enlarged field (yellow square) represents the GFP expression in the endothelial layer of the carotid artery. (**c**) STZ-induced diabetic TekCreERT2/DRG mice were subjected to a wire-induced injury or a wire injury plus liraglutide treatment (*n* = 4 in each group). The common carotid arteries were collected on day 28. Representative images show the immunofluorescence staining for GFP (green), α-SMA (red), and nuclei (blue) in wire-injured common carotid arteries. The enlarged field (yellow square) represents the co-localization of GFP expression in α-SMA-positive cells (white arrowheads) in the neointima. (**d**) Quantification of the number of GFP^+^SMA^+^ cells in the neointima area between groups. (**e**) Quantification of the neointima to media ratio between groups. * *P* < 0.05, analyzed by Student’s *t*-test.

Next, we examined whether cells of the endothelial lineage contribute to the process of neointima formation via endothelial-mesenchymal transition (EndMT) in vivo. We crossbred the TekCreERT2 mice with UBC-DsRed-emGFP reporter mice to obtain Tek-CreERT2;UBC-DsRed-emGFP (TekCreERT2/DRG) mice, in which endothelial-specific inducible Cre was driven by the Tek promoter in endothelial cells and in which the GFP reporter could be monitored as a marker of Cre recombination (Figure 2a). Before tamoxifen-induced Cre recombination, endothelial cells expressed the DsRed reporter gene. To determine whether endothelial cells were specifically labelled, we injected the TekCreERT2/DRG mice with tamoxifen at a dose of 20 mg/kg/day for five days and analyzed the GFP expression in the carotid artery (Figure 2b). After tamoxifen-induced Cre recombination, the DsRed gene cassette in Tek-expressing endothelial cells was deleted, as evidenced by the cellular expression of GFP (Figure 2b), which confirmed the specificity of genetic labelling in endothelial cells of TekCreERT2/DRG mice.

### 3.3. Endothelium-Derived Cells Contribute to Neointima Formation Following Artery Injury through EndMT in Diabetic Mice

To examine whether endothelium-derived cells contribute to the progression of neointima formation, we generated the model of wire injury-induced neointima formation in the diabetic TekCreERT2/DRG mice. TekCreERT2/DRG mice were pre-injected with tamoxifen to induce the genetic labelling of endothelial cells. Fourteen days after the final dose of tamoxifen, the mice were treated with STZ to induce diabetes. Wire-induced injury surgery was then performed at 21 days after STZ injection. Fasting glucose levels were measured to confirm the induction of diabetes in mice. Neointima hyperplasia was evident at 28 days post-injury (Figure 2c). Endothelium-derived cells with positive GFP staining were found to co-localize with the smooth muscle marker α-SMA in the neointima lesion, indicating that endothelium-derived cells contribute to the progression of neointima formation. Moreover, liraglutide treatment reduced the number of GFP^+^SMA^+^ cells in the neointima area (Figure 2d) as well as neointima formation (Figure 2e).

### 3.4. High Glucose Induces EndMT in HUVECs 

**Figure 3:**
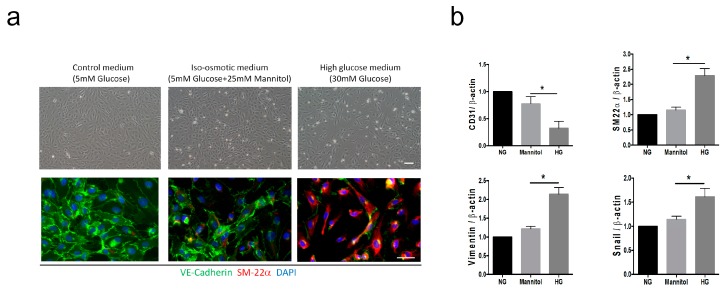
High glucose induces mesenchymal-like phenotypes in human umbilical venous endothelial cells (HUVECs). HUVECs were cultured in a control medium with a normal glucose level (NG, 5.5 mM of glucose), mannitol (M, 5.5 mM of glucose + 24.5 mM of mannitol), or high glucose (HG, 30 mM of glucose). (**a**) Representative bright-field images show the morphological changes in cells after 96 h of culture at different conditions. Scale bar represents 200 μm. The expression of VE-cadherin (green) and SM22α (red) in each group was analyzed using immunofluorescence staining. Nuclei were counterstained with DAPI (blue). HUVECs cultured in a HG medium showed a higher SM22α expression and a lower VE-cadherin expression compared to those in NG- or mannitol-treated groups. Scale bar represents 50 μm. (**b**) qRT-PCR analysis of the mRNA levels of CD31, SM22α, vimentin, and Snail in NG-, mannitol-, and HG-treated HUVECs. Data are shown as the mean ± SD from three independent experiments. * *P* < 0.05. The statistical significance was assessed by Kruskal–Wallis test.

We next tested the hypothesis that the HG condition promotes the transition of endothelial cells to the mesenchymal-like phenotype in vitro. We examined the morphological changes and gene expression of EndMT markers in HUVECs cultured with a control medium (C, 5 mM glucose), an iso-osmotic medium (M, 5 mM glucose plus 25 mM mannitol), and a HG medium (30 mM glucose). Mannitol was added to the iso-osmotic medium to match the osmolarity of the HG medium. We found that HUVECs cultured in the HG condition for 96 h showed distinctive spindle-like morphology compared to those in the control and M groups (Figure 3a). Endothelial cells lost their endothelial characteristics and gained a mesenchymal phenotype during the EndMT process, as reported previously [28]. Incubation in the HG condition for 96 h induced the expression of mesenchymal marker SM22α and inhibited the endothelial marker VE-cadherin in HUVECs, indicating that the HG condition triggers EndMT in vitro (Figure 3a). The qRT-PCR analysis confirmed that the HG condition induces a loss of the endothelial cell marker (CD31) and a gain of mesenchymal markers (vimentin, SM22α, and Snail) in HUVECs (Figure 3b).

### 3.5. High Glucose Induces mRNA and Protein Expression of EndMT Markers and Smad2-Snail Signaling in HUVECs 

**Figure 4:**
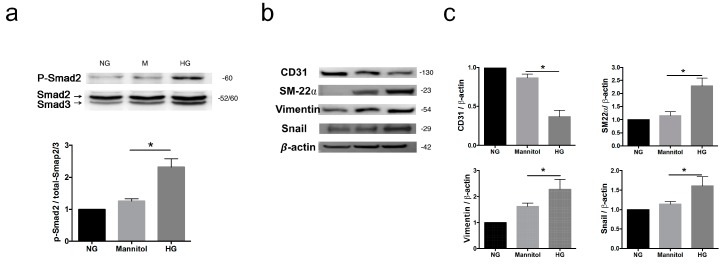
High glucose promotes EndMT in HUVECs. (**a**) Representative images of the western blot analysis of p-Smad2 protein levels. Total Smad2/3 served as the loading control. The quantification of protein levels is presented as mean ± SD from three independent experiments. (**b**) Representative images of the western blot analysis of CD31, SM22α, vimentin, and Snail protein levels in HUVECs treated with different conditions. (**c**) Quantification of the relative fold increase is shown as the mean ± SD from three independent experiments. * *P* < 0.05. The statistical significance was assessed by Kruskal–Wallis test.

The signal transduction molecule of activated-Smad2 (p-Smad2) was induced by HG, indicating an association between HG-induced EndMT via enhanced TGF-β signaling (Figure 4a). Western blot analyses also revealed that HG induced the protein expression of SM22α, vimentin, and Snail, and it decreased endothelial marker CD31 (Figure 4b,c). These results together indicate that HG induces EndMT in HUVECs.

### 3.6. Liraglutide Inhibits High Glucose-Induced EndMT in HUVECs via AMPK Pathway

Our results showed that liraglutide inhibits neointima formation and EndMT in diabetic mice in vivo (Figure 1 and Figure 2). We next aimed to delineate the underlying mechanisms of the liraglutide-mediated inhibition of HG-induced EndMT in vitro. The pro-inflammatory cytokine IL-1β has been shown to promote EndMT and mediate inflammatory signals in HG-induced EndMT [29,30]. To mimic the pro-inflammatory and hyperglycaemic status during neointima formation in diabetic mice, HUVECs were treated with HG, IL-1β, or HG+IL-1β for 24 h. 

**Figure 5:**
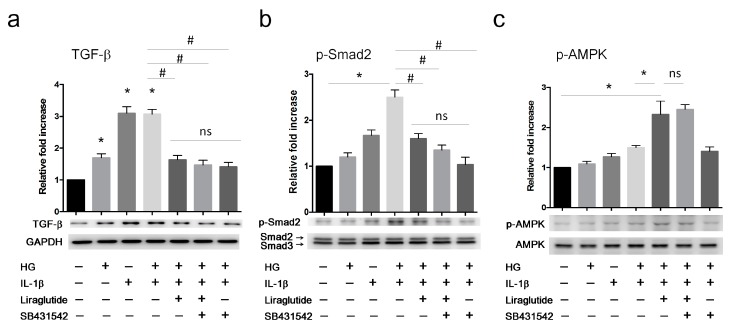
Liraglutide attenuates high glucose- and IL-1β-induced TGF-β signaling in HUVECs. HUVECs were cultured in the conditions indicated for 24 h. Cells were harvested for western blot analyses of the protein levels of (**a**) TGF-β and Glyceraldehyde 3-phosphate dehydrogenase (GAPDH) as an internal control, (**b**) p-Smad2 and total Smad2/3 as an internal control, and (**c**) p-AMPK and total AMPK as an internal control. Quantification of the relative fold increase is shown as the mean ± SD from three independent experiments. * *P* < 0.05 compared to saline control. # *P* < 0.05 compared to HG+IL-1β group. The statistical significance was assessed by Kruskal–Wallis test.

In vitro, we found that treatment with HG or IL-1β alone increased TGF-β expression (Figure 5a). The combination of HG+IL-1β synergistically induced the phosphorylation of Smad2 (Figure 5b). Moreover, treatment with liraglutide or ALK5 inhibitor SB431542, which blocks TGF-β receptor signaling, attenuated the effect of HG+IL-1β-mediated TGF-β and p-Smad2 upregulation. These observations suggested that HG, in combination with pro-inflammatory stimuli such as IL-1β, augments TGF-β signaling via ALK5 (Figure 5a,b). Interestingly, liraglutide increased the phosphorylation of AMPK in the HG+IL-1β group, whereas this was not altered in the presence of the ALK5 inhibitor (Figure 5c).

**Figure 6:**
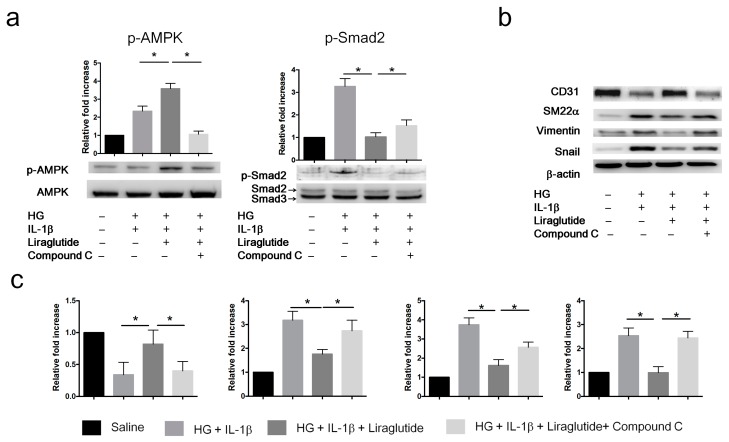
Liraglutide inhibits EndMT in HUVECs via the AMPK pathway. HUVECs were cultured in the following four conditions for 72 h: (1) Saline; (2) HG (30 mM glucose) plus IL-1β; (3) HG plus IL-1β and Liraglutide; (4) Liraglutide and Compound C. Cell lysates were collected for western blot analyses. (**a**) Western blot analysis of the protein levels of p-AMPKα and p-Smad2. Total AMPKα and Smad2/3 were used as loading controls for p-AMPK and p-Smad2, respectively. (**b**) Western blot analysis of the protein levels of CD31, SM22α, vimentin, and Snail in HUVECs treated with different conditions. Β-actin was used as the loading control. (**c**) Quantification of the relative fold increase is shown as the mean ± SD from three independent experiments. * *P* < 0.05. The statistical significance was assessed by Kruskal–Wallis test.

To further confirm whether liraglutide inhibits EndMT via the AMPK pathway, we treated the HUVECs with liraglutide (1 µg/mL) or with the AMPK inhibitor Compound C (1 µM) followed by exposure to HG+IL-1β for 72 h. Liraglutide enhanced p-AMPK and reduced p-Smad2 in HG+IL-1β-treated HUVECs (Figure 6a). The inhibition of AMPK by Compound C attenuated the liraglutide-mediated effects on signal transduction (p-AMPK and p-Smad2) and EndMT marker expression (CD31, SM22α, Snail, and vimentin) (Figure 6b,c). Taken together, these results indicate that liraglutide reverses HG+IL-1β-induced EndMT via the AMPK pathway. 

### 3.7. Liraglutide Activates Endothelial AMPK Signaling and Enhances Endothelial Recovery In vivo

**Figure 7:**
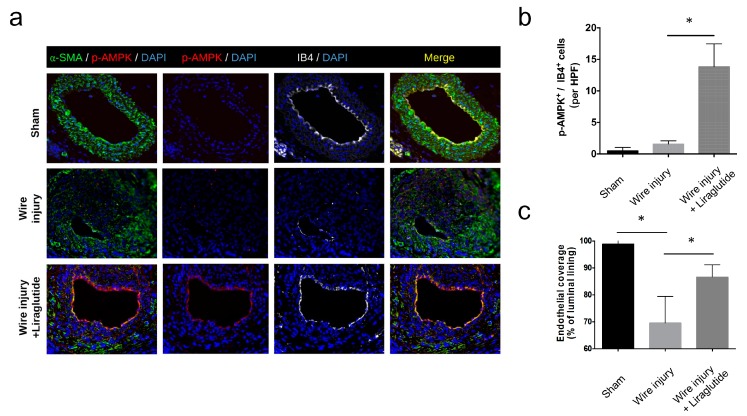
Liraglutide activates AMPK and enhances endothelial recovery in vivo. (**a**) STZ-induced diabetic C57BL/6 mice were subjected to a wire-induced carotid artery injury with or without liraglutide treatment. The expression of α-SMA (green) and p-AMPK (red) in the carotid arteries was analyzed by immunofluorescence staining. The endothelium was labelled by isolectin B4 (IB4, grey). Nuclei were stained with DAPI. (**b**) Number of p-AMPK^+^IB4^+^ endothelial cells per high-power field (HPF). (**c**) Percentage of endothelial coverage of the vascular lumen. Data are shown as mean ± SD, from three independent experiments. * *P* < 0.05. The statistical significance was assessed by Kruskal–Wallis test.

To further address whether liraglutide activates endothelial AMPK signaling in vivo, we examined the expression of pAMPK in the wire-injured carotid arteries from diabetic mice. We found that liraglutide treatment increased the levels of pAMPK (Figure 7a) in the isolectin B4 (IB4)-labelled endothelium (Figure 7b). In addition, liraglutide treatment increased endothelial coverage of the artery following the wire injury (Figure 7c). Together, these results indicated that liraglutide activated endothelial AMPK signaling, improved endothelial recovery, and reduced EndMT in vitro and in vivo.

## 4. Discussion

Our results show that HG condition-treated HUVECs acquired spindle morphology and mesenchymal markers with a loss of their original endothelial cell markers, which were in line with previous reports that HG induced a loss of endothelial phenotype in HUVECs [16,31]. These findings also confirmed that HG induced the EndMT process in HUVECs, which was in accordance with previously published results [15,21,32]. Inflammation and oxidative stress contribute to the EndMT process [9,14], but it is unclear how HG induces EndMT. The HG condition is reported to increase the expression of TGF-β [33]. Our findings revealed that the Snail-Smad signaling pathway was upregulated during HG-induced EndMT in HUVECs. Snail has been associated with the process of EndMT in previous studies [34,35]. The phosphorylation of Smad2 is a downstream event of the TGF-β signaling [36,37]. These findings suggest that upregulation of Smad signaling is associated with the HG-triggered EndMT process. 

The EndMT process is triggered by chronic hyperglycaemia, and EndMT was found to participate in diabetic cardiomyopathy, nephropathy, and retinopathy [11,38,39,40]. A higher restenosis rate is found in diabetic patients compared to the non-diabetic population even in the era of drug-eluting stents [41]. Several growth factors are proposed to promote vascular smooth muscle proliferation in HG conditions [42]. Medial vascular smooth muscle cells, circulating bone marrow-derived cells, and circulating stem/progenitor cells are all seen as the cellular origin of neointimal lesions [43,44,45]. Our findings revealed that endothelium-derived cells participate in neointima lesion formation through the EndMT process in diabetic mice, which is in line with a previous report demonstrating that endothelium-derived cells participate in neointima lesions through EndMT via activation of the TGF-β-Snail-Smad signaling pathway in biomechanical stress-induced pathological vascular remodeling of vein grafts [7]. Taken together, these findings might explain the worse outcome of diabetic patients in clinical practice.

Liraglutide, a new long-acting GLP-1 analogue, has recently been reported to possess a cardiovascular protective effect; however, the underlying molecular mechanism remained unclear. Several studies provided evidence of GLP-1 receptor expression on endothelial cells [46,47,48,49]. The GLP-1 receptor-mediated activation of the AMPK pathway by GLP-1 has been shown to attenuate diabetes-induced endothelial dysfunction and diabetic nephropathy [50,51,52]. In this study, our results demonstrated that liraglutide inhibits HG-induced Smad2 phosphorylation and EndMT via the AMPK pathway, suggesting that liraglutide mediates preservation of the endothelial phenotype and prevents hyperglycaemia-induced EndMT. Liraglutide activated the GLP-1 receptor to trigger AMPK via PKA-dependent phosphorylation of the LKB1 pathway in endothelial cells [47,48]. AMPK signaling plays an important role in the regulation of endothelial function and prevents HG-induced diabetic nephropathy, endothelial dysfunction, and impaired re-endothelisation [53,54,55]. Interestingly, AMPK signaling was also known to inhibit the TGF-β-mediated Smad2/3 pathway, as well as the epithelial-to-mesenchymal transition (EMT) [53,56,57]. In addition, AMPK signaling is essential for angiogenesis in response to hypoxic stress [58], which contributes to re-endothelisation [55]. Together, these findings suggest that the vascular protective role of liraglutide is mediated by the activation of the AMPK pathway in diabetic mice. 

In this study, we found that liraglutide treatment upregulated the expression of p-AMPK in the endothelium layer (Figure 7). In a previous report, Kushima et al. found that early liraglutide treatment suppressed the neointima formation through the stimulation of the endothelial AMPK-nitric oxide axis [47]. This finding suggested the possible involvement of liraglutide-mediated inhibition of vascular inflammation and stimulation of endothelial nitric oxide signaling in the context of liraglutide-induced suppression of EndMT in vivo. In vitro, we found that: (1) Liraglutide inhibits HG-induced EndMT and activates the AMPK pathway; (2) AMPK inhibition by Compound C abolished the liraglutide-mediation inhibition of HG-induced EndMT. In vivo, we confirmed that liraglutide inhibits HG-induced EndMT and activates the AMPK pathway. Therefore, it is logical to hypothesise that liraglutide-mediated AMPK signaling attribute to the liraglutide-mediated inhibition EndMT in vivo. However, further in vivo studies specifically targeting endothelial AMPK signaling might help to answer this question.

Eriksson et al. reported that Exendin-4 did not facilitate endothelial regeneration, but it did suppress neointimal hyperplasia [59]. There are two possibilities to explain the differences between ours and the findings by Eriksson et al. First, we used an STZ-induced diabetic mouse model. In line with our observation, Feng et al. recently demonstrated that the activation of GLP-1 receptor signaling promoted carotid vascular re-endothelisation only in the diabetic condition but not in the non-diabetic condition in a rat model [46]. Second, in our study, we used liraglutide instead of Exendin-4. Liraglutide was shown to provide cardiovascular protective effects in patients with diabetes [17]. The different potency and half-life of these compounds may explain the pharmacological differences between the two drugs.

**Figure 8:**
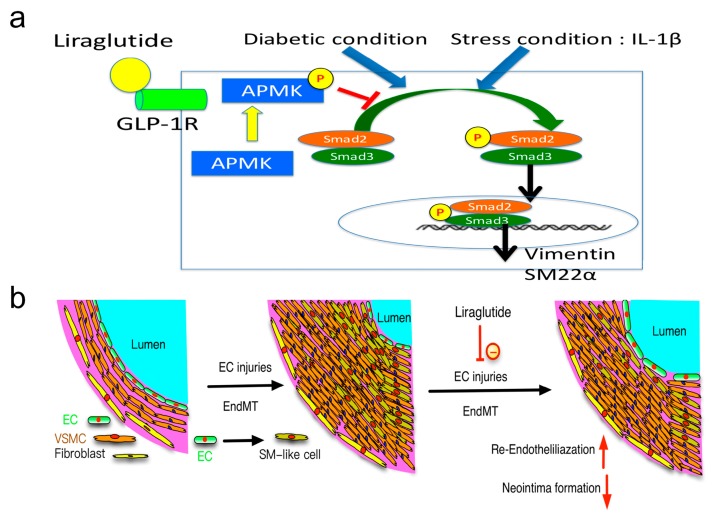
Liraglutide attenuates diabetes-associated neointima formation and pathogenic EndMT via the AMPK pathway. (**a**) The proposed underlying mechanisms of the therapeutic effects of liraglutide on the attenuation of a diabetes-associated vascular injury. Upon diabetic conditions such as hyperglycaemia in combination with pro-inflammatory stimulation by IL-1β, endothelial cells undergo the pathogenic EndMT process with increased mesenchymal marker expression (vimentin and SM22α). Liraglutide, a GLP-1 agonist, activates downstream AMPK signaling via GLP-1R and attenuates diabetic insult-induced EndMT in endothelial cells. (**b**) In vivo, endothelium-derived smooth muscle-like cells contribute to the progression of neointima formation after a wire-induced carotid artery injury. Reduced neointima formation in diabetic mice upon liraglutide treatment may be partially attributed to the reduced EndMT process.

The wire injury-induced endothelial damage triggered the EndMT process and occurred in the early phase of neointima formation [7]. The exact origin of cells in the neointima has been explored in previous studies. Medial smooth muscle cells, bone marrow-derived cells, and stem cells in adventitia are found to participate in neointima formation [43,44,45]. Our study evidenced that endothelium-derived cells participate in the formation of neointima in diabetic mice. Systemic GLP-1 agonist or analogues are effective in attenuating neointima formation by inhibiting the inflammatory process [22,60]. The pro-inflammatory response also initiates and promotes the EndMT process. The damage of endothelial cells may trigger endothelial cells to transdifferentiate into mesenchymal-like cells with a loss of their initial endothelial functions and characteristics, which delays the re-endothelisation process. In clinical practice, diabetic patients show a higher incidence of in-stent restenosis [41]. Our findings demonstrate that the EndMT process was inhibited through acceleration of the re-endothelisation process mediated by liraglutide, which may explain the underlying mechanism of GLP-1 agonist-mediated cardiovascular protective effects (Figure 8). 

This study has several limitations. First, this study did not investigate the optimal dosage of liraglutide for maximizing the vascular protective effects in the carotid artery injury model. However, previous studies have demonstrated that a relatively high dose of liraglutide (1000 µg/kg/day) offered adequate protective effects in mouse models of atherosclerosis [24]. The dosage of liraglutide used in the present study (1000 µg/kg/day) was therefore identical to that of the previous study [24]. Second, the diabetic mice used in the present study were 12 weeks old with a diabetic duration of five weeks. However, the clinical situation was not well reflected in our study setting. It would thus be inappropriate to directly extrapolate these results to the clinical setting. Third, the attenuation of EndMT was mediated by the inhibition of AMPK in this study; however, the precise signaling pathway(s) through which liraglutide exerted its therapeutic effects were not elucidated. Fourth, in our study, we did not analyze the cardiac functions of mice and systemic parameters such as lipid profiles and inflammation cytokines. Though liraglutide inhibited IL-1β-triggered EndMT in vitro, we did not exclude the possibility that liraglutide attenuates the EndMT process through the inhibition of the systemic inflammation process in vivo. Fifth, we demonstrated that liraglutide inhibited HG+IL-1-β-induced TGF-β protein in HUVECs, whereas the effect of the secretion and activation of the TGF-β protein was not analyzed in our in vitro experiments. Whether liraglutide-mediated AMPK signaling altered the secretion and activation of TGF-β await further investigation.

## 5. Conclusions

Liraglutide is currently used in diabetic patients for controlling serum glucose levels. Our study demonstrated that (1) the HG condition promotes the process of EndMT in HUVECs; (2) cells of endothelial origin are involved in neointima formation in a wire-injured carotid artery in diabetic mice; (3) liraglutide reduces neointima formation and accelerates re-endothelisation in diabetic mice; and (4) liraglutide inhibits HG-induced EndMT via the AMPK pathway in vitro and in vivo. These results together suggest a beneficial effect of liraglutide on neointima formation through the inhibition of EndMT in a diabetic mouse model (Figure 8a,b). Thus, our findings may explain, at least in part, the therapeutic mechanisms of liraglutide in diabetic patients and encourage the possible use of liraglutide in diabetic patients with coronary artery disease, in the prevention of restenosis, and promoting re-endothelialisation.

## Supplementary Materials

The following are available online at https://www.mdpi.com/2073-4409/8/6/589/s1, Table S1: Primers used in this study. Table S2: Serum glucose levels of the mice at different stages between the experimental groups.
