# Supplementary material for: Liraglutide Inhibits Endothelial-to-Mesenchymal Transition and Attenuates Neointima Formation after Endovascular Injury in Streptozotocin-Induced Diabetic Mice
Source: Cells. 2019 Jun 14;8(6):589. doi: 10.3390/cells8060589 (PMC6628350; doi:10.3390/cells8060589)
Supplement: Supplementary file 1 [file cells-08-00589-s001.pdf]

Supplementary Table S1. Primers used in this study

Gene	specific oligonucleotide primers for target sequences
CD31	F: 5’-CAACGAGAAAATGTCAGA-3’
	R: 5’-GGAGCCTTCCGTTCTAGAGT-3’
Vimentin	F: 5’-GCAAAGATTCCACTTTGCGT-3’
	R: 5’-GAAATTGCAGGAGGAGATGC-3’
SM22alpha	F: 5’-CTGAGGACTATGGGGTCATC-3’
	R: 5’-TAGTGCCCATCATTCTTGGT-3’
Snail	F: 5’-GCTGCAGGACTCTAATCCAGA-3’
	R: 5’-ATCTCCGGAGGTGGGATG-3’
Beta-actin	F: 5’-CCAACCGCGAGAAGATGA-3’
	R: 5’-CCAGAGGCGTACAGGGATAG-3’

Supplementary Table S2

Serum glucose levels of the mice at different stages between the experimental groups

	Sham	Wire-injury	Wire-injury with Liraglutide treatment	*p*
Baseline	131.5 +/- 5.7	137.4 +/- 4.8	132 +/- 5.2	0.907
Day 0	564.5 +/- 23.5	546.7 +/- 21.4	568.8 +/- 30.2	0.876
Day 14	402.2 +/- 10.3	410.5 +/- 14.8	432.2 +/- 29.6	0.206
Day 28	452.4 +/- 11.7	449.9 +/- 13.2	484.0 +/- 34.6	0.106

The data is shown as the mean ± SEM from all groups (n = 10, in each group) and analysed with one-way non-parametric ANOVA test. Day 0, 14, 28 indicated the days post wire-injury.
